# A Novel Nomogram to Predict Resectable Gastric Cancer Based on Preoperative Circulating Tumor Cell

**DOI:** 10.14309/ctg.0000000000000561

**Published:** 2023-01-24

**Authors:** Feng Xia, Qiao Zhang, Elijah Ndhlovu, Mingyu Zhang, You Zou

**Affiliations:** 1 Department of Hepatic Surgery, Tongji Hospital, Tongji Medical College of Huazhong University of Science and Technology, Wuhan, Hubei, China;; 2 Zhongshan People's Hospital Affiliated to Guangdong Medical University, Guangdong, China;; 3 Department of Digestive Medicine, Tongji Hospital of Tongji Medical College of Huazhong University of Science and Technology, Wuhan, China;; 4 Gastrointestinal Surgery Center, Tongji Hospital of Tongji Medical College of Huazhong University of Science and Technology, Wuhan, Hubei, China.

**Keywords:** circulating tumor cells, resectable gastric cancer, nomogram, prognosis

## Abstract

**INTRODUCTION:**

Circulating tumor cells (CTCs) have been suggested to have an important prognostic role in gastrointestinal tumors. We developed a preoperative CTC-based nomogram to predict the prognosis of patients with resectable gastric cancer after surgery and established a risk stratification system based on the nomogram.

**METHODS:**

From January 2012 to June 2017, we screened 258 patients with gastric cancer treated with surgery from one center as the training cohort and 133 patients with gastric cancer treated with surgery from another as the validation cohort, screened prognostic factors for the training cohort using univariate and multivariate Cox risk proportional models, created predictive overall survival (OS) and a recurrence-free survival (RFS) nomogram, and plotted the receiver operating characteristic curve and calibration curve for this nomogram in the training and validation cohorts. Risk score stratification was performed according to the nomogram, and OS curves were plotted for the low, medium, and high-risk groups using the Kaplan-Meier method.

**RESULTS:**

The CTC positivity rate was 78.5% in all patients. CTC, TNM stage, and Ki-67 were the prognostic factors affecting OS and RFS after gastric cancer surgery. The nomogram consisted of these 3 variables. In the training group, the area under the curve of the nomogram for OS at 1, 3, and 5 years was 0.918, 0.829, and 0.813, respectively, and the area under the curve for RFS was 0.900, 0884, and 0.839, respectively. There was a statistically significant difference in OS among the low, medium, and high-risk groups according to the risk stratification system constructed from nomogram scores (*P* < 0.001).

**DISCUSSION:**

Two nomograms based on preoperative CTC were established to predict OS and RFS after resectable gastric cancer. The 2 nomograms had good discrimination and calibration and significant stratification ability of the risk stratification system established according to them.

## INTRODUCTION

Gastric cancer is the fourth most common cancer globally, with the number of people suffering from it rising each year, and the second leading cause of cancer death worldwide. To date, surgical resection is one of the most effective treatments of resectable cancers, and research on the prognosis of patients after surgery is ongoing (1,2). Although many researchers have tried to use some new biomarkers to predict the postoperative outcome of patients with gastric cancer in recent years, the clinical prediction of patients' postoperative outcomes is still inadequate (3,4). Therefore, there is an urgent need to discover new and more representative markers and predictive models to improve postoperative survival or recurrence prediction.

Circulating tumor cells (CTCs) are tumor cells disseminated in the peripheral blood of patients with tumors. The current technology for monitoring CTCs is mature, and the presence of CTCs has been detected in different cancer types. The presence of CTCs symbolizes a poor prognosis for patients (5,6). A meta-analysis by Rahbari et al (7) concluded that CTC plays an essential role in predicting the prognosis of patients with colorectal cancer. Currently, CTC is essential to predict the prognosis of patients with gastric cancer after surgery. No investigator has used CTCs to build predictive models to predict overall and recurrence-free survival (RFS) after surgery for resectable gastric cancer. This study aims to establish a nomogram based on CTC—a marker of importance—and validate it with a validation group and finally use the nomogram to establish a risk scoring system to stratify the management of patients with resectable gastric cancer.

## METHODS

### Patient selection

Data were collected from one center from 258 patients with resectable gastric cancer who underwent surgery between January 2012 and June 2017 and were called the training group. Data from 133 patients with resectable gastric cancer who underwent surgical treatment were collected from another center. We used strict inclusion criteria, and the inclusion criteria were as follows: (i) postoperative tumor histopathology determined to be gastric cancer by 2 experienced pathologists; (ii) patients' TNM stage was strictly determined according to the eighth edition of the American Joint Committee on Cancer, mainly by postoperative pathology; (iii) American Society of Anesthesiologists (ASA) score ≤II; (iv) Eastern Oncology Collaborative Group (ECOG) physical status score ≤2; (v) normal liver and kidney function; (vi) the tumor was found for the first time and had not undergone endoscopic mucosal resection or endoscopic submucosal dissection before surgery; and (vii) postoperative pathology was reported by 2 pathologists to be negative for cut margins. Patients at both centers underwent a uniform standardized treatment protocol. In principle, patients with preoperative lymph node metastases are recommended to undergo chemotherapy. However, the centers fully respect the patient's wishes, and the final decision on whether to undergo preoperative chemotherapy largely depends on the patient's wishes. This retrospective study was approved by the Ethics Committee of Tongji Hospital, Tongji Medical College, Huazhong University of Science and Technology, and the Ethics Committee of Zhongshan People's Hospital and followed the Declaration of Helsinki.

### Surgery

The tumor is assessed for invasion of surrounding organs and vital blood vessels and for distant metastases by a specialist team of gastroenterologists and imaging experts. When the tumor invades the hilar region, pancreas, aorta, and mesenteric root vessels, it is an indication for surgical nonresection (8). Preoperative tests such as red blood cell count and liver and kidney function tests are routinely performed to assess the suitability for surgery. The location of the tumor in the stomach determines the extent of gastrectomy: total gastrectomy for tumors growing in the upper third; total gastrectomy for tumors growing in the body of the stomach or distal gastrectomy, depending on the case; and distal gastrectomy for tumors growing in the distal part of the stomach (sinus). An experienced surgical team performs all surgical procedures.

### Detection of CTCs

One week before the gastrectomy, we first took approximately 5 mL of peripheral blood and standardized the removed samples according to the company's instructions. CTC was detected using the Cyttel method (Jiangsu, China), which consists of the negative immunomagnetic particle method and immunofluorescence *in situ* hybridization. The former mainly uses immunomagnetic particles as a carrier to remove leukocytes from blood and isolate rare cells *in vitro* by the principle of antigen-antibody reaction combined with the centrifugation technique. The samples are fixed on slides, dehydrated with ethanol, dried, and hybridized with Chromosome Adhesion Probe 1 and Chromosome Adhesion Probe 1. Finally, 4-diamidino-2-phenylindole was added to stain the closed specimens, and CTCs were observed and counted under fluorescence microscopy. Based on previous studies and multicenter discussions, it defined CTC counts ≥1 as CTC-positive (9,10).

### Follow-up

We followed up with all patients every 3 months during the first year after discharge, and every 6 months after the first year. Imaging examinations, such as abdominal ultrasound, enhanced computed tomography, and abdominal magnetic resonance imaging, and laboratory tests, such as liver function and kidney function, were performed at each follow-up visit. For patients undergoing surgery, overall survival (OS) was defined as the time from postoperative day 1 to death; RFS was defined as the time from postoperative day 1 to the first imaging finding of tumor or metastasis. The follow-up period was ended October 30, 2021.

### Data analysis

The χ^2^ or Fisher exact test was used to compare all categorical variables. The Kaplan-Meier method was used for the comparison of OS and RFS of diseases. The log-rank test was used for the comparison of survival rate. Univariate and multivariate analyses were performed for OS and RFS after gastrectomy using the Cox proportional model. Variables with *P* < 0.05 in univariate analysis were included in multivariate regression analysis. Receiver operator characteristic curves were used to assess the model's discrimination, and calibration curves were used to assess the calibration of the model. A risk scoring system was then established based on the total score of each patient in the training cohort (sum of the scores of each variable given by the column line graph), and patients were divided into 3 risk groups—low, medium, and high—with a similar number of cases in them.

All statistical analyses were performed using SPSS 25.0 (IBM, Armonk, NY), and *P* values <0.05 (both sides) were considered statistically significant. R software (version 4.0.5; R Project for Statistical Computing, Vienna, Austria) was used to generate the Kaplan-Meier curves, receiver operator characteristic curves, and calibration curves. X-tile software (version 3.6.1) determined the cutoff value of the scores in the training cohort. The sample size was calculated using PASS (version 11.0) before conducting the study. We set 2-sided alpha = 0.05 and beta = 0.1 (sample power = 0.9).

## RESULTS

### Characteristics of the patients with gastric cancer in the training cohort and validation cohort

The inclusion and exclusion flow charts for the training and validation cohorts are shown in Supplementary Figure 1 (see Supplementary Digital Content 1, http://links.lww.com/CTG/A907). There were a total of 258 patients in the training cohort and 133 patients in the validation cohort; in the training cohort, there were 215 male patients (83.3%), 111 (43.0) older than 60 years, and 50 CTC-positive patients (19.4%); in the validation group, there were 111 male patients (83.5%), 65 (65) older than 60 years (48.9%), and 34 patients (25.6%) were CTC-positive. Nearly 20 percent of the patients at both centers received preoperative chemotherapy. None of the variables was statistically different between the 2 groups (Table 1).

**Table 1.** Baseline characteristics of patients with gastric cancer undergoing gastrectomy in the training cohort and validation cohort (n = 391)

	Training cohort (n = 258)	Validation cohort (n = 133)	*P* value
Sex, n (%)			1.000
Male	215 (83.3)	111 (83.5)	
Female	43 (16.7)	22 (16.5)	
Age, n (%)			0.285
<60 yr	147 (57.0)	68 (51.1)	
≥60 yr	111 (43.0)	65 (48.9)	
ASA, n (%)			0.446
I	217 (84.4)	116 (87.9)	
II	40 (15.6)	16 (12.1)	
ECOG PS, n (%)			1.000
0	203 (78.7)	105 (78.9)	
1	55 (21.3)	28 (21.1)	
Depth of tumor invasion, n (%)			0.388
T1	48 (18.6)	28 (21.1)	
T2	23 (9.0)	14 (10.5)	
T3	123 (47.7)	71 (53.4)	
T4	64 (24.8)	20 (15.0)	
N status, n (%)			0.124
N0	62 (24.0)	25 (18.8)	
N1	70 (27.1)	28 (21.1)	
N2	57 (22.1)	37 (27.8)	
N3	69 (26.7)	43 (32.3)	
TNM stage, n (%)			0.432
I stage	86 (33.3)	50 (37.6)	
II stage	88 (34.1)	48 (36.1)	
III stage	84 (32.6)	35 (26.3)	
Preoperative chemotherapy			0.529
No	196 (76.0)	105 (78.9)	
Yes	62 (24.0)	28 (21.1)	
Differentiation			0.436
Well + moderately	52 (20.2)	32 (24.1)	
Poorly	206 (79.8)	101 (75.9)	
Operation performed			0.718
Total gastrectomy	77 (29.8)	45 (33.8)	
Distal gastrectomy	168 (65.1)	82 (61.7)	
Others	13 (5.1)	6 (4.5)	
Extent of lymphadenectomy			0.636
D1	51 (19.8)	29 (21.8)	
D1 + D2/D3	207 (80.2)	104 (78.2)	
Lymphatic invasion			0.653
No	72 (27.9)	40 (30.0)	
Yes	186 (72.1)	93 (70.0)	
Tumor number, n (%)			0.556
Single	248 (96.1)	130 (97.7)	
Multiple	10 (3.9)	3 (2.3)	
Tumor size, n (%)			0.054
<5 cm	114 (44.2)	73 (54.9)	
≥5 cm	144 (55.8)	60 (45.1)	
Vascular invasion, n (%)			0.369
No	92 (35.7)	41 (30.8)	
Yes	166 (64.3)	92 (69.2)	
Ki-67, n (%)			0.522
<50%	134 (51.9)	74 (55.6)	
≥50%	124 (48.1)	59 (44.4)	
CTC, n (%)			0.193
Negative	208 (80.6)	99 (74.4)	
Positive	50 (19.4)	34 (25.6)	

TNM stages are according to the American Joint Committee on Cancer, Eighth Edition.

ASA, American Society of Anesthesiologists; CTC, circulating tumor cell; ECOG, Eastern Cooperative Oncology Group; PS, performance status.

### Impact of CTC on patient prognosis

**Figure 1.**
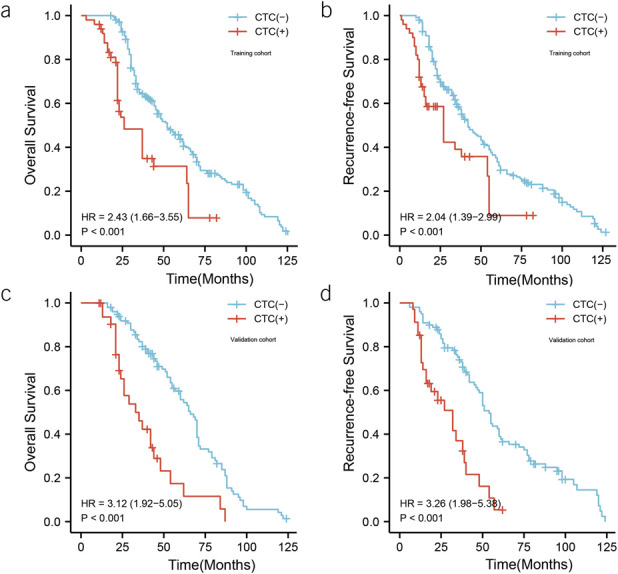
Impact of positive or negative circulating tumor cells (CTCs) on overall survival and recurrence-free survival of patients after surgery. (**a**) Overall survival of CTC(+) vs CTC(−) in the training cohort. (**b**) Recurrence-free survival of CTC(+) vs CTC(−) in the training cohort. (**c**) Overall survival of CTC(+) vs CTC(−) in the validation cohort. (**d**) Recurrence-free survival of CTC(+) vs CTC(−) in the validation cohort.

In the training group, the prognosis of CTC(+) patients was worse than that of CTC(−) patients. The 1-, 3-, and 5-year OS of CTC(+) patients was 94.1%, 47.2%, and 23.0%, respectively, with a median survival time of 26.0 months; the 1-, 3-, and 5-year OS of CTC(−) patients was 100.0%, 64.7%, and 44.0%, respectively, with a median survival was 52.0 months, with statistically significant differences in OS curves between the 2 groups (hazard ratio [HR] = 1.43 [1.66–3.55], *P* < 0.001). The 1-, 3-, and 5-year RFS for CTC(+) patients was 70.6%, 38.3%, and 8.8%, respectively, with a median time to relapse of 27.0 months; CTC(−) patients had RFS of 98.6%, 58.8%, and 33.5% at 1, 3, and 5 years, respectively, with a median time to recurrence of 42.0 months, with statistically significant differences in RFS curves between the 2 groups (HR = 2.04 [1.39–2.99], *P* < 0.001). In the validation group, CTC(+) patients were also worse than CTC(−) patients in overall survival and RFS (Figure 1).

### Univariate and multivariate Cox regressions as prognostic factors in patients with gastric cancer

**Figure 2.**
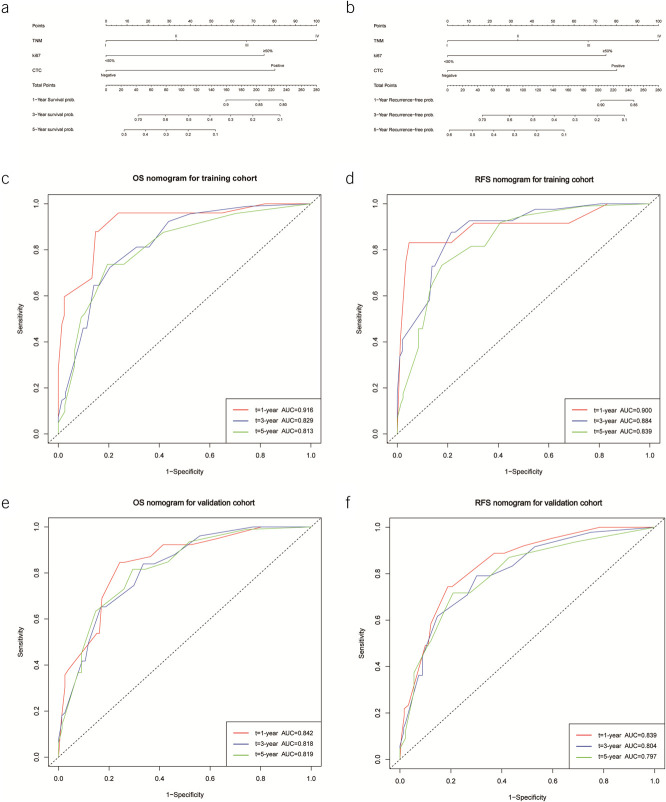
Column plots of predicted overall survival (OS) and recurrence-free survival (RFS) based on the results of multivariate Cox regression analysis and plotting of time-dependent receiver-operating characteristic (ROC) curve. (**a**) and (**b**) Nomograms of predicted OS and RFS, respectively. (**c**) and (**d**) 1-, 3-, and 5-year ROC curves in the training cohort and their area under the curve (AUC), respectively. (**e**) and (**f**) 1-, 3-, and 5-year ROC curves in the validation cohort and their AUC, respectively.

In the training cohort, we included the statistically significant screening obtained from the univariate Cox regression analysis into the multifactorial regression analysis based on *P* < 0.05. TNM stage (HR = 1.678 [1.238–1.881]), Ki-67 (HR = 2.150 [1.512–2.876]), differentiation (HR = 1.645 [1.234–2.121]), extent of lymphadenectomy (HR = 0.568 [0.389–0.912]), lymphatic invasion (HR = 1.674 [1.112–2.431]), and CTC (HR = 2.345 [1.756–3.567]) were considered as prognostic factors affecting patients' OS (Table 2); TNM stage (HR = 1.432 [1.198–1.744]), Ki-67 (HR = 1.879 [1.438–2.510]), differentiation (HR = 1.687 [1.152–2.342]), extent of lymphadenectomy (HR = 0.668 [0.588–0.973]), lymphatic invasion (HR = 1.777 [1.287–2.347]), and CTC (HR = 2.213 [1.675–2.887]) were also considered as prognostic factors affecting patients' RFS (Table 3), and a nomogram was created based on these 3 variables obtained from the training cohort (Figure 2a and b)

**Table 2.** Univariate and multivariate analyses of overall survival in patients with gastric cancer who underwent gastrectomy in the training cohort

	Univariate analysis			Multivariate analysis		
	*P* value	HR	95% confidence interval	*P* value	HR	95% confidence interval
Sex Male/female	0.964	1.009	0.678–1.470			
Age >60 yr/≤60 yr	0.157	0.812	0.588–1.083			
ASA II/I	0.747	1.063	0.733–1.543			
ECOG PS 1/0	0.916	0.982	0.699–1.379			
TNM stage	<0.001	1.666	1.458–1.903	<0.001	1.678	1.238–1.881
Preoperative chemotherapy Yes/no	0.048	0.891	0.562–0.988	0.073	0.788	0.751–1.087
Tumor size >5.0 cm/≤5.0 cm	0.023	1.483	1.098–2.116	0.312	1.189	0.734–1.921
Differentiation Poorly/well + moderately	0.013	1.588	1.134–2.238	0.001	1.645	1.234–2.121
Operation performed Total gastrectomy/distal gastrectomy	0.132	1.224	0.875–1.532			
Extent of lymphadenectomy D1 + D2/D1	0.032	0.772	0.518–0.891	0.003	0.568	0.389–0.912
Lymphatic invasion Yes/No	0.040	1.586	1.087–2.331	0.015	1.674	1.112–2.431
Vascular invasion Yes/no	0.297	0.857	0.641–1.145			
Ki-67 ≥50%/<50%	<0.001	2.354	1.611–2.252	<0.001	2.150	1.512–2.876
CTC +/−	<0.001	2.391	1.633–3.502	<0.001	2.345	1.756–3.567

TNM stages are according to the American Joint Committee on Cancer, Eighth Edition.

ASA, American Society of Anesthesiologists; CTC, circulating tumor cell; ECOG, Eastern Cooperative Oncology Group; HR, hazard ratio; PS, performance status.

**Table 3.** Univariate and multivariate analyses of recurrence-free survival in patients with gastric cancer who underwent gastrectomy in the training cohort

	Univariate analysis			Multivariate analysis		
	*P* value	HR	95% confidence interval	*P* value	HR	95% confidence interval
Sex Male/female	0.940	0.986	0.677–1.435			
Age >60 yr/≤60 yr	0.133	0.803	0.603–1.069			
ASA II/I	0.475	0.870	0.595–1.274			
ECOG PS 1/0	0.638	1.084	0.776–1.514			
TNM stage	<0.001	1.500	1.316–1.710	<0.001	1.432	1.198–1.744
Preoperative chemotherapy Yes/no	0.102	0.781	0.483–1.158			
Tumor size >5.0 cm/≤5.0 cm	0.052	1.325	0.998–1.760			
Differentiation Poorly/well + moderately	0.042	1.842	1.108–2.732	0.022	1.687	1.152–2.342
Operation performed Total gastrectomy/distal gastrectomy	0.288	1.132	0.779–1.645			
Extent of lymphadenectomy D1 + D2/D1	0.012	0.881	0.645–0.934	0.034	0.668	0.588–0.973
Lymphatic invasion Yes/no	0.030	1.377	1.013–2.431	0.013	1.777	1.287–2.349
Vascular invasion Yes/no	0.829	1.033	0.770–1.386			
Ki-67 ≥50%/<50%	<0.001	2.617	1.942–3.528	<0.001	1.879	1.438–2.510
CTC +/−	<0.001	2.015	1.374–2.957	<0.001	2.213	1.675–2.887

TNM stages are according to the American Joint Committee on Cancer, Eighth Edition.

ASA, American Society of Anesthesiologists; CTC, circulating tumor cell; ECOG, Eastern Cooperative Oncology Group; HR, hazard ratio; PS, performance status.

### Test the discrimination and calibration of the nomogram

In the training cohort, a time-dependent receiver-operating characteristic (ROC) curve showed that the patients' areas under the curve (AUCs) for 1-, 3-, and 5-year OS were all more than 0.80, at 0.916, 0.829, and 0.813, respectively. Similarly, the time-dependent ROC showed that the patients' AUCs for 1-, 3-, and 5-year RFS were 0.900, 0.884, and 0.839, respectively. AUC of 0.900, 0.884, and 0.839, respectively; using the validation group, essentially all 1-, 3-, and 5-year AUCs for OS and RFS were more than 0.800.

**Figure 3.**
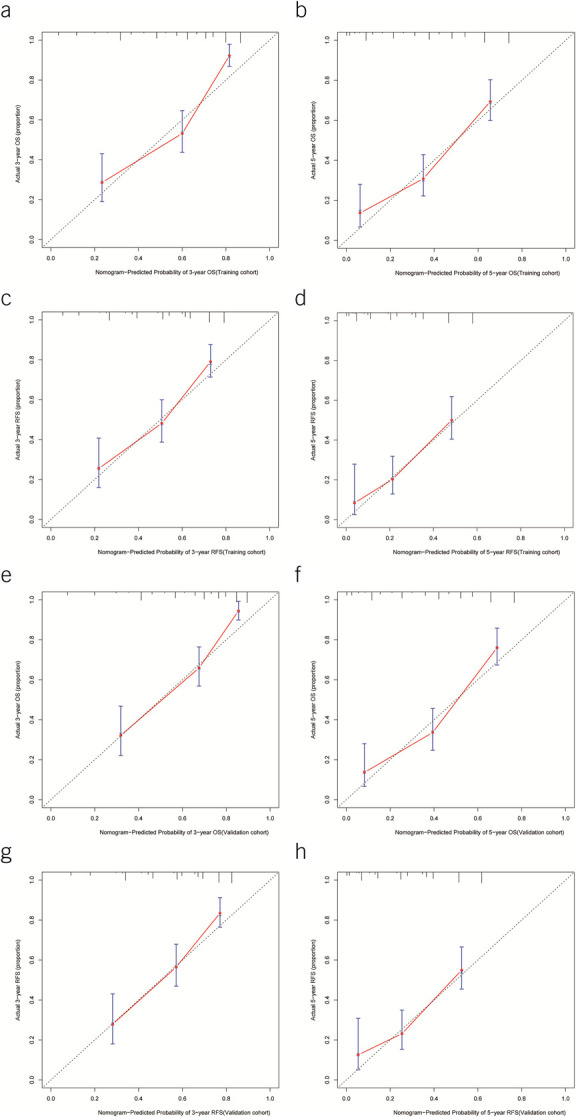
Calibration curves of the predicted nomogram for overall survival (OS) and recurrence-free survival (RFS). (**a**) and (**b**) 1-, 3-, and 5-year calibration of the nomogram for OS in the training cohort. (**c**) and (**d**) 1-, 3-, and 5-year calibration of the nomogram for RFS in the training cohort. (**e**) and (**f**) 1-, 3-, and 5-year calibration of the nomogram for OS in the validation cohort. (**g**) and (**h**) 1-, 3-, and 5-year calibration of the nomogram for RFS in the validation cohort.

The calibration curves were plotted to evaluate the 2 newly created models. In the training cohort, the actual 3- and 5-year OS and RFS are in good agreement with the 3- and 5-year OS and RFS predicted by the nomogram (Figure 3a–d). In the validation cohort, the actual 3- and 5-year OS and RFS are also in good agreement with the 3- and 5-year OS and RFS predicted by the nomogram (Figure 3e–h).

### Generate a risk scoring system based on the nomogram

**Figure 4.**
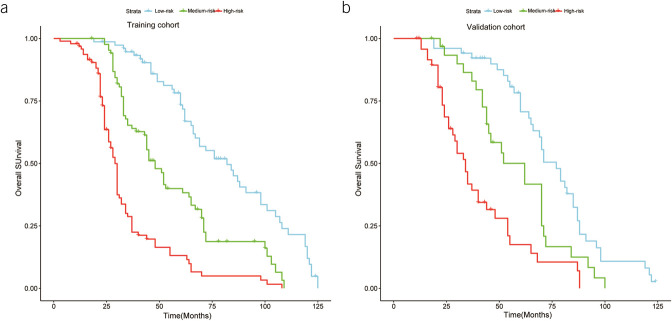
Overall survival in the training and validation sets for the low, medium, and high-risk groups.

In the training cohort, we used X-tile software to determine the cutoff values based on the total score given to each patient in the nomogram and classified the patients into low, medium, and high groups. In the low-risk group, the patients' 1-, 3-, and 5-year OS was 100.0%, 94.6%, and 73.4%, respectively; the median survival time was 82.0 months. In the medium-risk group, the patients' 1-, 3-, and 5-year OS was 100.0%, 64.0%, and 38.3%, respectively, with a median survival time of 48.0 months; in the high-risk group, patients had an OS of 96.9%, 28.7%, and 11.5%, respectively, for 1, 3, and 5 years, with a median survival time of 29.0 months, with a significant prognostic difference between the 3 groups (*P* < 0.01). The validation group also had a significant prognostic difference between the low, medium, and high-risk groups (Figure 4).

## DISCUSSION

Surgical resection is the first-line treatment of gastric cancer, especially resectable gastric cancer, which is often curable. However, the 5-year survival rate after surgery is still poor, and disease progression such as recurrence can occur. Some researchers (11–13) have used tumor markers such as CEA and CA19-9 to predict patients after surgery, but these markers are limited and often negative. In the meantime, some investigators have developed models to predict the prognosis of patients with gastric cancer after surgery, but they are often unsatisfactory, with relatively low predictive efficacy (14,15). Therefore, there is a need for effective biomarkers to predict postoperative survival and the risk of recurrence and predictive models and stratification of postoperative patients for more refined management.

CTCs are a type of tumor cell present in the circulating blood system (16). Recently, an increasing number of articles have demonstrated the prognostic role of CTCs in predicting patients with different types of cancers (17–19). Our study assessed the prognostic role of CTCs in patients with resectable gastric cancer after surgery and showed that positive CTCs were associated with a poorer prognosis in both OS and RFS, and the same results were found in the validation group. Meanwhile, multivariate Cox regression analysis also indicated that CTC was a significant prognostic factor affecting OS and RFS. Nowadays, studies on the prognostic role of CTC in gastrointestinal tract tumors are actively being conducted. Zhang et al (20) compared the changes in CTC values in preoperative and postoperative patients with gastric cancer and concluded that increased postoperative CTC might increase hematogenous recurrence; Ito et al (21) concluded that recurrence is closely related to the number of patients with CTC , and a meta-analysis by Gao et al (22) similarly suggested that CTC could identify patients with worse prognosis of gastric cancer and showed more robust and more stable predictive value in advanced disease. Thus, considerable evidence also suggests that CTC is important in predicting patient prognosis, and we are the first study to use CTC to predict postoperative prognosis in patients with resectable gastric cancer and develop a prognostic model based on CTC.

All patients with early gastric cancer (Tis or T1aN0M0) included in our cohort were treated surgically. However, the current treatment modalities for early gastric cancer are mainly surgical and endoscopic, which are somewhat controversial. Pourmousavi et al (23) analyzed a sizeable population-based database from 1998 to 2014 and found an increasing trend in endoscopic treatment of superficial gastric cancer compared with surgery. Patients who underwent endoscopic treatment vs surgery had comparable long-term cancer-specific mortality. Similarly, a meta-analysis by Sun et al (24) identified 9 retrospective studies that showed endoscopic resection might be a feasible and safe treatment strategy compared with gastrectomy. At present, careful endoscopic testing is needed to ensure good results and validation in large-scale randomized controlled studies from different countries. However, the trend toward endoscopic treatment of early gastric cancer is slowly expanding.

We established a nomogram for predicting OS and RFS after resectable gastric cancer. Previously, Yu and Zhang (25) constructed a nomogram to predict the prognosis of gastric cancer in young patients with a C-index of 0.688, which is lower than the C-index of our nomogram. Additionally, other researchers have constructed nomograms to predict gastric cancer survival by combining multiple genes (15,26), and Zhu et al (27) predicted early death in metastatic gastric cancer by Surveillance, Epidemiology, and End Results. However, these studies suffer from insufficient sample size and sample size from public databases. Our nomograms were composed of TNM stage, Ki-67, and CTC and were validated by the validation group. TNM staging is a vital staging system with different prognostic survival assessments for patients with different stages of gastric cancer. However, TNM has certain limitations. For example, the treatment at different stages is not agreed upon, and combining it with CTC can provide the opportunity to go beyond the individualized risk assessment determined by TNM staging only (28). There is also some inter-relationship with the TNM stage, with stage III and IV tumors exhibiting greater ease of detection of CTCs. Notably, Ki-67 also has a solid predictive role, and in our multivariate Cox regression analysis, the variable Ki-67 was highly weighted in the model. Ki-67 has also received attention from researchers in recent years because it has long been recognized as a value-added marker for tumors and also has strong potential as a diagnostic marker for gastrointestinal tract tumors, as suggested by Yang et al (29). The Ki-67 index was negatively correlated with tumor differentiation, and the CTC index was not correlated with the Ki-67 index. The 2 markers were independent of each other. Ki-67 also has a vital role in predicting the prognosis of patients with cancer, and a meta-analysis by Jung-Soo Pyo (30) showed that a high Ki-67 index was associated with a poorer prognosis of patients with gastric cancer. Therefore, the prediction model built by the above 3 variables has excellent clinical efficacy, and this is the first CTC-based nomogram for postoperative gastric cancer.

Based on the nomogram, we constructed a risk stratification system that can classify all postoperative patients with gastric cancer into 3 risk groups, i.e., low, medium, and high-risk groups; it can be seen from the survival curve that the prognosis difference between the 3 risk groups is very obvious. We can take more refined management in our clinical work for different risk groups, especially for the high-risk group. The 5-year survival rate for patients in the high-risk group was only 11.5%, so we can consider alternative treatments to reduce the pain caused by surgery or adjuvant treatments for the high-risk group, both preoperatively and postoperatively.

This study has several limitations. First, this study is a retrospective study with confounding factors; second, we have only 1 external datum as validation, and more external data may be needed for validation in the future; third, our center is a large tertiary referral center and may have patients from a different source than other centers, so there is some selection bias. Moreover, the process of some adjuvant treatments, such as postoperative chemotherapy, was not studied for the patients, which may affect the results.

In conclusion, we constructed a nomogram for predicting postoperative OS and RFS in patients with gastric cancer based on CTC, a vital marker that is highly practical and has a high degree of differentiation and accuracy. The risk stratification system established by the column line chart can guide clinicians to personalize the treatment of postoperative patients with gastric cancer and provide a basis for the clinical management of high-risk patients.

## CONFLICTS OF INTEREST

**Guarantor of the article:** You Zou, MD.

**Specific author contributions:** F.X.: conceptualization, methodology, software, and writing—original draft preparation. Q.Z.: conceptualization and methodology. E.N.: data curation and writing—reviewing and editing. M.Z.: visualization and investigation. Y.Z.: writing—reviewing and editing.

**Financial support:** None to report.

**Potential competing interests:** None to report.

**Study Highlights**

**WHAT IS KNOWN**

✓ Circulating tumor cells (CTC) are an important predictive marker in gastrointestinal tumors.

**WHAT IS NEW HERE**

✓ We constructed a model to predict postoperative gastric cancer based on CTC.

## References

[1] Joshi SS Badgwell BD. Current treatment and recent progress in gastric cancer. CA Cancer J Clin 2021;71(3):264–79. 33592120 10.3322/caac.21657 PMC9927927

[2] Thrift AP El-Serag HB. Burden of gastric cancer. Clin Gastroenterol Hepatol 2020;18(3):534–42. 31362118 10.1016/j.cgh.2019.07.045 PMC8859863

[3] Hu L Zhang P Sun W . PDPN is a prognostic biomarker and correlated with immune infiltrating in gastric cancer. Medicine (Baltimore) 2020;99(19):e19957. 32384443 10.1097/MD.0000000000019957 PMC7220208

[4] Wu D Zhang P Ma J . Serum biomarker panels for the diagnosis of gastric cancer. Cancer Med 2019;8(4):1576–83. 30873760 10.1002/cam4.2055 PMC6488129

[5] Thanh Huong P Gurshaney S Thanh Binh N . Emerging role of circulating tumor cells in gastric cancer. Cancers (Basel) 2020;12(3):695. 32183503 10.3390/cancers12030695 PMC7140068

[6] Zhou J Ma X Bi F . Clinical significance of circulating tumor cells in gastric cancer patients. Oncotarget 2017;8(15):25713–20. 28147337 10.18632/oncotarget.14879 PMC5421964

[7] Rahbari NN Aigner M Thorlund K . Meta-analysis shows that detection of circulating tumor cells indicates poor prognosis in patients with colorectal cancer. Gastroenterology 2010;138(5):1714–26.e13. 20100481 10.1053/j.gastro.2010.01.008

[8] Lim JS Yun MJ Kim MJ . CT and PET in stomach cancer: Preoperative staging and monitoring of response to therapy. Radiographics 2006;26(1):143–56. 16418249 10.1148/rg.261055078

[9] Chen Z Wang T Chen C . Circulating tumor cell is a clinical indicator of pretransplant radiofrequency ablation for patients with hepatocellular carcinoma. J Oncol 2021;2021:7776389. 34712326 10.1155/2021/7776389 PMC8548160

[10] Zhang Q Xia F Mo A . Guiding value of circulating tumor cells for preoperative transcatheter arterial embolization in solitary large hepatocellular carcinoma: A single-center retrospective clinical study. Front Oncol 2022;12:839597. 35664772 10.3389/fonc.2022.839597 PMC9159764

[11] Feng F Tian Y Xu G . Diagnostic and prognostic value of CEA, CA19-9, AFP and CA125 for early gastric cancer. BMC Cancer 2017;17(1):737. 29121872 10.1186/s12885-017-3738-y PMC5679342

[12] Jing R Cui M Ju S . The changes and clinical significance of preoperative and postoperative serum CEA and CA19-9 in gastric cancer. Clin Lab 2020;66(4). doi:10.7754/Clin.Lab.2019.190732. 32255307

[13] Shen M Wang H Wei K . Five common tumor biomarkers and CEA for diagnosing early gastric cancer: A protocol for a network meta-analysis of diagnostic test accuracy. Medicine (Baltimore) 2018;97(19):e0577. 29742692 10.1097/MD.0000000000010577 PMC5959440

[14] Bando E Ji X Kattan MW . Development and validation of pretreatment nomogram for disease-specific mortality in gastric cancer-A competing risk analysis. Cancer Med 2021;10(21):7561–71. 34628732 10.1002/cam4.4279 PMC8559461

[15] Liu Y Wu J Huang W . Development and validation of a hypoxia-immune-based microenvironment gene signature for risk stratification in gastric cancer. J Transl Med 2020;18(1):201. 32410620 10.1186/s12967-020-02366-0 PMC7226948

[16] Ferreira MM Ramani VC Jeffrey SS. Circulating tumor cell technologies. Mol Oncol 2016;10(3):374–94. 26897752 10.1016/j.molonc.2016.01.007 PMC5528969

[17] Kulasinghe A Hughes BGM Kenny L . An update: Circulating tumor cells in head and neck cancer. Expert Rev Mol Diagn 2019;19(12):1109–15. 31680565 10.1080/14737159.2020.1688145

[18] Nanduri LK Hissa B Weitz J . The prognostic role of circulating tumor cells in colorectal cancer. Expert Rev Anticancer Ther 2019;19(12):1077–88. 31778322 10.1080/14737140.2019.1699065

[19] Piñeiro R Martínez-Pena I López-López R. Relevance of CTC clusters in breast cancer metastasis. Adv Exp Med Biol 2020;1220:93–115. 32304082 10.1007/978-3-030-35805-1_7

[20] Zhang Q Shan F Li Z . A prospective study on the changes and clinical significance of pre-operative and post-operative circulating tumor cells in resectable gastric cancer. J Transl Med 2018;16(1):171. 29925382 10.1186/s12967-018-1544-1 PMC6011408

[21] Ito H Sato J Tsujino Y . Long-term prognostic impact of circulating tumour cells in gastric cancer patients. World J Gastroenterol 2016;22(46):10232–41. 28028372 10.3748/wjg.v22.i46.10232 PMC5155183

[22] Gao Y Xi H Wei B . Association between liquid biopsy and prognosis of gastric cancer patients: A systematic review and meta-analysis. Front Oncol 2019;9:1222. 31850190 10.3389/fonc.2019.01222 PMC6901923

[23] Pourmousavi MK Wang R Kerdsirichairat T . Comparable cancer-specific mortality of patients with early gastric cancer treated with endoscopic therapy vs surgical resection. Clin Gastroenterol Hepatol 2020;18(12):2824–32.e1. 32389885 10.1016/j.cgh.2020.04.085

[24] Sun K Chen S Ye J . Endoscopic resection versus surgery for early gastric cancer: A systematic review and meta-analysis. Dig Endosc 2016;28(5):513–25. 26701862 10.1111/den.12596

[25] Yu C Zhang Y. Development and validation of prognostic nomogram for young patients with gastric cancer. Ann Transl Med 2019;7(22):641. 31930042 10.21037/atm.2019.10.77 PMC6944578

[26] Bai Y Wei C Zhong Y . Development and validation of a prognostic nomogram for gastric cancer based on DNA methylation-driven differentially expressed genes. Int J Biol Sci 2020;16(7):1153–65. 32174791 10.7150/ijbs.41587 PMC7053317

[27] Zhu Y Fang X Wang L . A predictive nomogram for early death of metastatic gastric cancer: A retrospective study in the SEER Database and China. J Cancer 2020;11(18):5527–35. 32742500 10.7150/jca.46563 PMC7391207

[28] Mavroudis D. Circulating cancer cells. Ann Oncol 2010;21(Suppl 7):vii95–100. 20943650 10.1093/annonc/mdq378

[29] Yang Y Li J Jin L . Independent correlation between Ki67 index and circulating tumor cells in the diagnosis of colorectal cancer. Anticancer Res 2017;37(8):4693–700. 28739773 10.21873/anticanres.11874

[30] Pyo JS Kim NY. Meta-analysis of prognostic role of Ki-67 labeling index in gastric carcinoma. Int J Biol Markers 2017;32(4):e447–53. 28561880 10.5301/ijbm.5000277

